# Preparation of Immobilized Lipase Based on Hollow Mesoporous Silica Spheres and Its Application in Ester Synthesis

**DOI:** 10.3390/molecules24030395

**Published:** 2019-01-22

**Authors:** Zhe Dong, Meng-Ying Jiang, Jie Shi, Ming-Ming Zheng, Feng-Hong Huang

**Affiliations:** 1 Oil Crops Research Institute, Chinese Academy of Agricultural Sciences, Key Laboratory of Oilseeds Processing, Ministry of Agriculture, Oil Crops and Lipids Process Technology National &Local Joint Engineering Laboratory, Hubei Key Laboratory of Oil crops Lipid Chemistry and Nutrition, Wuhan 430062, China; dongzhehehe@163.com (Z.D.); shijie@caas.cn (J.S.); 2 Department of Chemistry, Wuhan University, Wuhan 430072, China; mokall@163.com; 3 Datang Gongyi Power Co., Ltd., Zhengzhou 451200, China

**Keywords:** immobilized lipase, hydrophobic modification, enzymatic esterification, phytosterols esters, response surface methodology

## Abstract

In this study, *Candida rugosa* lipase (CRL) was immobilized into modified hollow mesoporous silica (HMSS) materials with different hydrophobicity. Among propyl-(C_3_), phenyl-(C_6_), octyl-(C_8_), and octadecyl-(C_18_) modified HMSS as well as native HMSS, taking advantage of more hydrophobic microenvironment, the HMSS-C_18_-CRL showed exceptional performance in enzymatic esterification reaction. Using the novel HMSS-C_18_ with immobilized CRL (HMSS-C_18_-CRL), we investigated the esterification of phytosterols with polyunsaturated fat acid (PUFA) in a solvent-free system for the production of phytosterols esters. Response surface methodology (RSM) was applied to model and optimize the reaction conditions, namely, the enzyme load (5–25%), reaction time (10–110 min), molar ratio of α-linolenic acid (ALA)/phytosterols (1:1–7:1) and represented by the letters E, T, and M respectively. Best-fitting models were successfully established by multiple regressions with backward elimination. The optimum production was achieved at 70 min for reaction time, 20% based on the weight of substrate for enzyme loading, and 5.6:1 for ALA/phytosterols molar ratio. Under optimized conditions, a conversion of about 90 ± 2% was achieved. These results indicated that HMSS-C_18_-CRL demonstrates to be a promising catalyst and can be potentially applied in the functional lipid production.

## 1. Introduction

Enzymes are versatile as biocatalysts for the green synthesis of chemicals and pharmaceuticals due to the milder catalytic conditions, higher catalytic efficiency, and fewer side effects as compared to chemical catalysts [1,2,3]. Lipase (triacylglycerol ester hydrolases, EC 3.1.1.3) is indispensable among the family of enzymes on account of the remarkable catalytic performance in hydrolysis, esterification, transesterification and alcoholysis [4]. However, free lipase has not been widely applied on an industrial scale for the challenges associated with its poor durability and recyclability. The immobilization of lipase could not only overcome those difficulties, but also endow the biocatalysts with special properties, such as higher activity, modulation of the enzyme selectivity and decrease of the reaction inhibition [4].

So far, there are four main methods for immobilization of enzyme pertaining to physical adsorption, covalent binding, cross-linked enzyme aggregates and entrapment. Among those methods, the immobilization of adsorption is the most important and useful method and thus many studies about immobilization based on adsorption were conducted with different novel supports [2]. Significant efforts have been made to develop various and advanced supports for the immobilization of lipase, including porous silica [5,6,7,8], graphene oxides [9], metal-organic frameworks [10] and covalent organic frameworks [11]. Among the numerous possible enzyme immobilization carriers, mesoporous silica is a particularly attractive material for the immobilization of enzymes since it possesses an ordered pore structure, narrow pore size distribution [12] and a large surface area [13,14], it is chemically and mechanically stable [15] and resistant to microbial attack and it can be chemically modified with various functional groups [16,17]. In addition, hollow mesoporous silica spheres (HMSS) possess with large surface area, low density and large inner void space [18], which provides sufficient space and ideal accommodation for lipases [3]. In general, lipases show interfacial activation, where the active site of the lipase is covered by an amphiphilic α-helical loop [19]. Once the hydrophobic surface is activated it will give easier access to the active site for substrates [20]. Taking this into consideration, hydrophobic supports have the advantage of enhancing the activity of immobilized lipases. However, HMSS are more hydrophilic due to the presence of many hydroxyl groups. Many HMSS modifications will improve the surface wettability and adsorption capacity of the supports. Jin et al. investigated the catalytic performance of immobilized PCL with different kinds of hydrophobic/hydrophilic surfaces, finding that the most hydrophobic silica nanomaterial demonstrated the highest activity [5]. Mohammad et al. designed octadecylalkyl modified mesoporous silica nanoparticles, adjusting the particle and pore sizes and providing an elaborate analysis of the nanomaterial’s properties [20]. However, few reports about immobilization of lipase based on modified hollow silica nanomaterials and comprehensive evaluations of their performance in esterification applications have been published.

Phytosterols, which are widely distributed in nuts, vegetable oils, seeds, cereals, and beans, have been found to have the ability to lower the plasma cholesterol concentration due to their similar structure to cholesterol [21,22,23]. However, the usage of phytosterols as a dietary supplement is limited by their low solubility in edible oil and high melting point [24]. Dietary α-linolenic acid (ALA), an n−3 polyunsaturated fatty acid, is a member of the essential fatty acids group. It plays an important role in the development of the brain and the retina [25], but free ALA is susceptible to oxidation because of its multiple unsaturated bonds. Therefore, esterification of phytosterols with ALA can not only overcome the poor solubility and low bioactivity of free phytosterols, but also endow the phytosterol esters with more activity as food ingredients. Benefiting from their significant medicinal and health-care functions, the effective and rapid synthesis of phytosterol esters has attracted the attention of many pharmaceutical and food companies [26]. So far, phytosterol esters have mainly been synthesized by chemical methods [27]. In the drive toward green and sustainable methodologies, enzymatic esterification of phytosterols, especially by immobilized lipases, has become more and more attractive [28,29,30,31,32]. However, a number of the earlier studies were carried out in the presence of organic solvents, which were environmentally unfriendly and the resulting products were prone to requiring multiple product separation and purification steps as well as containing solvent residues. Solvent free systems, offering greater volumetric production and solvent savings, are more attractive for food industry applications. The absence of solvent facilitates downstream processing since fewer components would be present at the end of the reaction, thus minimizing the production cost [33]. In line with the desire for a green and sustainable environment, a solvent-free catalyst system is desirable for the synthesis of phytosterol esters and commercial applications.

**Scheme 1:**
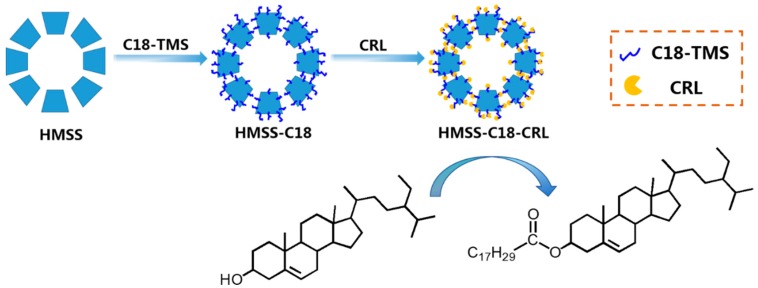
Schematic overview of preparation procedures of HMSS-C_18_-CRL and biocatalytic synthesis of phytosterol esters. C_18_-TMS: trimethoxyoctadecylsilane; CRL: *Candida rugosa* lipase.

In this work, we immobilized *Candida rugosa* lipase (CRL) on different hydrophobic modified hollow mesoporous silica sphere (HMSS) materials to prepare efficient immobilized enzymes for the esterification of phytosterols with ALA in a solvent-free system (Scheme 1). This lipase was selected because it had been successfully utilized for esterification of phytosterols [29,33] and the hollow mesoporous structure was chosen because it exhibits more advantages in terms of mass diffusion and transport compared with conventional mesoporous materials due to its larger pores and cavity volumes and spherical morphology. The catalytic activity of modified HMSS has been enhanced greatly with the lipase ‘lid’ opening. Conditions for the esterification of ALA with phytosterols in a solvent-free system were modeled and optimized by response surface methodology (RSM).

## 2. Results and Discussion

### 2.1. Characterizations of HMSS, HMSS-C_18_ and HMSS-C_18_-CRL

**Figure 1:**
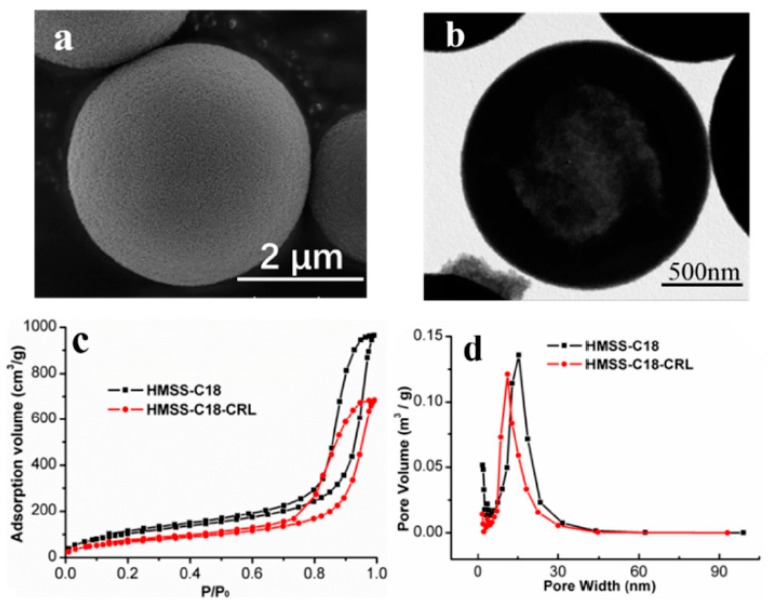
(**a**) SEM image of HMSS (**b**) TEM image of HMSS (**c**) nitrogen adsorption–desorption isotherms of HMSS-C_18_ and HMSS-C_18_-CRL (**d**) pore size distribution curves of HMSS-C_18_ and HMSS-C_18_-CRL.

To gain better insight into the materials, structural characterizations were carried out. The SEM images in Figure 1a demonstrate that the HMSS exhibited a monodisperse, uniform and spherical morphology with an average diameter of 3.8 μm. The hollow structure of the HMSS was evidenced by the TEM images (Figure 1b). It can be seen that the diameter of the cavities was about 1.0 μm and the wall thickness of the silica layer is about 0.3 μm.

**Figure 2:**
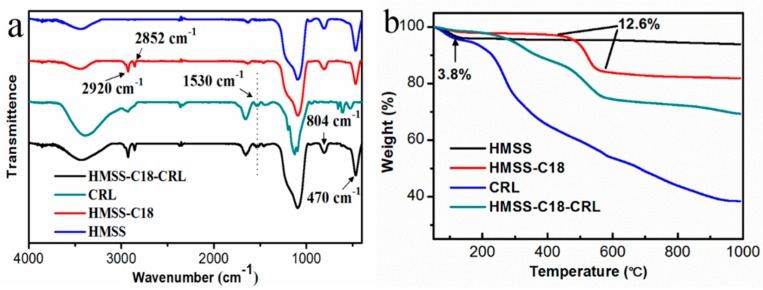
(**a**) The FT-IR (**b**) TGA of HMSS, HMSS-C_18_, CRL and HMSS-C_18_-CRL.

The nitrogen adsorption-desorption isotherms of HMSS-C_18_ and HMSS-C_18_-CRL shown in Figure 1c,d and Table 1 indicate that they all possess good mesoporous structures (showing typical type IV and H_1_ isotherm patterns) and the average pore size was about 12 nm. Compared with a reported study [34], the surface area and pore volume of HMSS-C_18_ and HMSS-C_18_-CRL were all decreased. It is therefore indicated that the C_18_ chain was grafted successfully onto HMSS-C_18_, which was firmly loaded with numerous lipase molecules. The FT-IR spectra shown in Figure 2a verify these results. Comparing the spectra of HMSS-C_18_ and HMSS, the peaks at 2852 cm^−1^ and 2920 cm^−1^ could be assigned to C-H symmetric and asymmetric stretching vibrations and the peak at 1530 cm^−1^ could be assigned to the -N-H bending vibration in the spectra of CRL and HMSS-C_18_-CRL. It was noteworthy that the pore size of each material remained unchanged, so we can interpret these results as indicating that the lipase was distributed uniformly on the HMSS-C_18_ without clusters and the appropriate pore volume and size offered sufficient space for the reactants to come in and out.

The thermal behaviour of HMSS, HMSS-C_18_, CRL and HMSS-C_18_-CRL was investigated by TGA analysis in a nitrogen atmosphere. As shown in Figure 2b, the weight of CRL and HMSS was decreased slightly at 50–100 °C owing to the loss of water, while the weight of HMSS-C_18_ and HMSS-C_18_-CRL was almost unchanged at this stage. This indicated that HMSS-C_18_ and its immobilized lipase were more hydrophobic or the water absorption capacity was weakened after the modification, so the activity of HMSS-C_18_-CRL in the esteridication can be improved. The weight loss of HMSS-C_18_ at 450 °C to 500 °C was ascribed to octadecyl chain pyrolysis, accounting for about 12.6% of the total weight of HMSS-C_18_. In summary, the modification and enzyme immobilization on HMSS were successful. Meanwhile, the mesoporous hollow structure, large pore volume and appropriate pore size lends HMSS-C_18_ as an ideal support for the immobilization of lipase and application in catalytic reactions.

### 2.2. Evaluation of Immobilized CRLs with Different Hydrophobicities.

**Figure 3:**
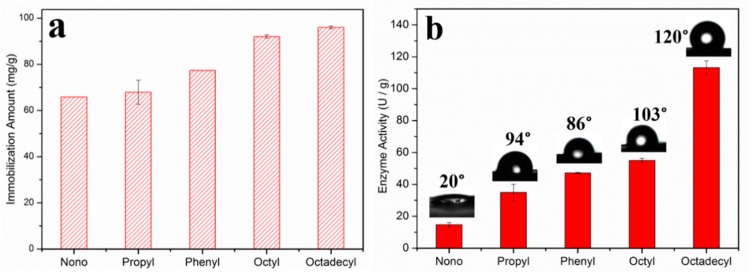
(**a**) The amount of the immobilized lipase and (**b**) the water contact angle and apparent activity of HMSS and modified HMSS.

In this study, enzyme immobilization was carried out by physical adsorption on the carrier. A schematic illustration was shown in Scheme 1. Among the various immobilization methods, adsorption on solid carriers has been the most common since it is simple and will not change the native structure of the enzyme. Ethanol prewetting of hydrophobic carriers was employed as previously reported to avoid enzyme adsorption only on the outer shell of the carrier [35]. In order to investigate the effrect of the surface hydrophobicity of HMSS on the activity and amount of lipase immobilized, four immobilized lipases with different surface modifications were prepared and compared. The water contact angle, loading amount and hydrolytic activity of different immobilized lipases with surface modification are shown in Figure 3. One more thing worth mentioning is that the larger the water contact angle is, the more hydrophobic the surpport is. It could be seen from Figure 3b that as the chain length increased, the water contact angle became larger, namely the more hydrophobic the support surface was, however, as the hydrophobicity of the support increased, the amount of immobilized lipase wasn’t very different but the apparent activity increased, which was similar to the result reported in a previous work [36]. We could deduce that the modification of supports has little effect on the adsorption, while the hydrophobicity of supports may influence the biocatalytic activity by enhancing the lipase ‘lid’ opening and thus providing easier access to the active site. As CRL immobilized on HMSS-C_18_ showed the maximum apparent activity, it was chosen for further study.

**Figure 4:**
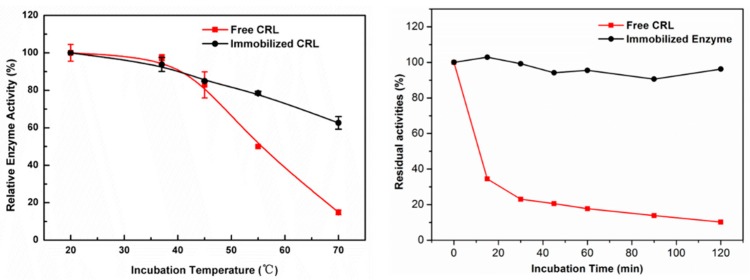
Relative enzyme activity (thermal stability) of free and immobilized CRL after (**a**) incubation at different temperature for 30 min (**b**) incubation at 55 °C.

Given the fact that the lipase is utilised at high tempertures, the thermal stabilty of an immobilized lipase is therefore significant [37]. The thermal stabilty of HMSS-C_18_-CRL and free CRL was studied by the p-NPP method and the best reaction temperature was investigated. The activity profiles of free and immobilized CRL incubated for 30 min in the absence of substrate at different temperatures are presented in Figure 4. 

As shown in Figure 4a, when the temperature increased above 35 °C, the relative activity of the free lipase decreased faster than the immobilized one. The free CRL retained only 65.5% residual activity, while immobilized lipase retained 82.3% of its initial activity at 50 °C. More obvious at 70 °C, the free CRL retained only 14.9% residual activity while immobilized CRL was found to retain 62.6% of its initial activity. To exclude any difference resulting from the reaction temperature, the thermal stability experiment was conducted at 37 °C which was obtained from the cross point in Figure 4a. As shown in Figure 4b, the huge disparity was more distinct after the incubation in 55 °C for different times. The activity of free CRL dropped sharply, with only 30% residual activity after incubation in 55 °C for 20 min but the activity of HMSS-C_18_-CRL remained almost unchanged with remarkable catalytic activity. Prolonging the incubation time, the free CRL was inactivated and the immobilized lipase still maintained a high 90% residual activity. Taking these data into account we postulate that HMSS-C_18_ may serve as a stronger shield to safeguard the lipase from deactivation and this makes HMSS-C_18_ very promising for application in the immobilization and stabilization of lipases.

### 2.3. Enzymatic Esterification of Phytosterols with ALA in a Solvent-Free System

#### 2.3.1. Effect of Temperature on the Degree of Esterification (DE)

**Figure 5:**
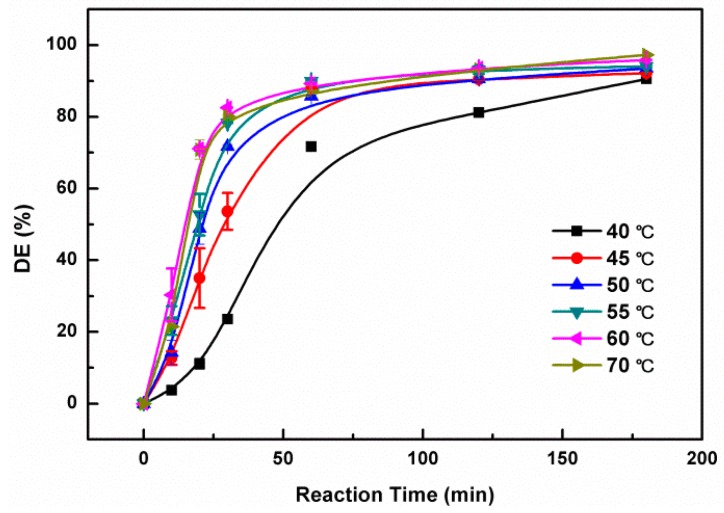
Time course of DE in solvent-free system at different temperature with an enzyme loading of 15.4% and 1:4 molar ratio of phytosterols to ALA.

Temperature is one of the crucial factors for the synthesis of phytosterol esters. In Figure 5, solvent-free esterification experiments at different temperatures were conducted with 15% (based on the weight of phytosterols) enzyme loading, and a 1:4 molar ratio of phytosterols to ALA, respectively. As the temperature increased from 40 to 70 °C, the initial rate of the reaction as well as the DE value increased and then all the DE time courses at different temperature seem to exhibit an asymptotic approach to equilibrium as the reaction time is prolonged. In general, the increase in temperature can improve the solubility of phytosterols and reduce the viscosity of the reaction system, resulting in enhancement of the reaction rate by making the interactions between the enzyme and substrates easier. However, high temperatures result in deactivation of the CRL which would shorten the reuse time of the immobilized enzyme. Therefore, in this study, 50 °C was selected as an optimal temperature for the reaction, because in the range of 50 to 70 °C, there was no significant difference in DE at longer times (>60 min) and the immobilized lipase retained more activity at 50 °C.

#### 2.3.2. Effect of Substrate Molar Ratio on DE

**Figure 6:**
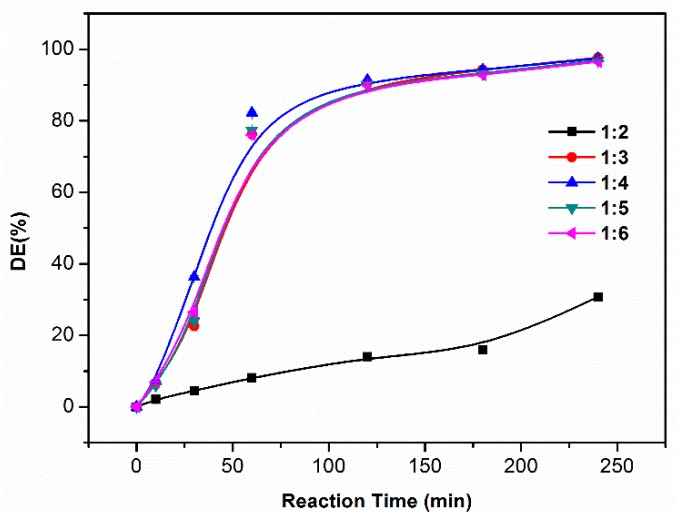
Time course of DE in solvent-free system at different molar ratios with enzyme loading of 15.4% and temperature of 50 °C.

Taking the cost factor and the separation of products into consideration, it is significant to optimize the substrate molar ratio and thus the content of phytosterols was held constant while the molar ratio of phytosterols to ALA ranged from 1:2 to 1:6. As shown in Figure 6, the DE was increased little by little at the molar ratio was 1:2 with a DE of 30%, which might stem from the small volume of reaction and the low solubility of phytosterols. When the molar ratio was risen to 1:3 and 1:4, the DE increased sharply to about 90% at 120 min. However, as the content of ALA continues to increase the DE remained stable. We deduced that the concentration of catalyst and phytosterol was decreased and thus the contact between substrate and enzyme was decreased, resulting in the stable DE value. In all, the substrate molar ratio of 1:4 was presumed to be the optimized one in the solvent-free catalyst system.

#### 2.3.3. Effect of Enzyme Loading on DE

**Figure 7:**
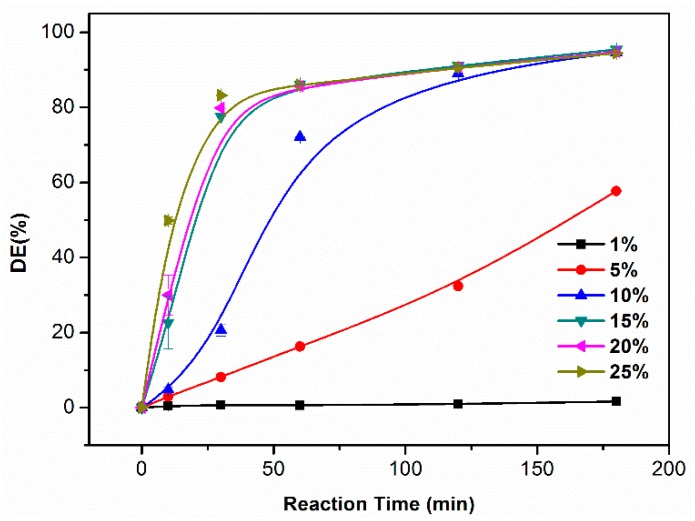
Time course of DE in a solvent-free system at different enzyme loadings with a molar ratio of 1:4 and temperature of 50 °C.

It is generally accepted that the more catalyst added, the higher the catalytic efficiency is within a certain range. The effect of enzyme loading was investigated over a range varying from 1% to 25% and the results are shown in Figure 7. The DE was increased as the enzyme loading increased to 15%, achieving the higher DE of 90%, and the DE or catalytic efficiency remained steady even if more enzyme was added. Benefitting from the fuller and more rapid ccontact between substrate and enzyme with increasing enzyme loading, the enzymatic catalytic reaction proceeded well. Considering the conversion of phytosterols esters and the cost of the enzyme, the enzyme loading of 15% was selected to be optimal amount added.

#### 2.3.4. Response Surface Methodology Analysis.

The degrees of esterification for different experimental parameters are given in Table 2. A five-level, three factorial central composite design of reaction time (X_1_), enzyme loading (X_2_), and molar ratio (X_3_) was investigated. The predicted conversion (Y) could be expressed in terms of the coded factors as follows:

Y = 82.74 + 7.43X_1_ + 9.82X_2_ + 6.63X_3_ − 4.12X_1_X_2_ − 1.63X_1_X_3_ − 3.69X_1_^2^ − 4.74X_2_^2^ − 3.45X_3_^2^ (1)

The predicted responses were most suitably described with a reduced cubic model (modified model) with backward elimination, and were seen to be closely correlated to the experimental values in Table 2. The fit of the models was evaluated by the coefficients of determination (R^2^) and an ANOVA test for lack of fit (Table 3). The very small *p* values (<0.0001) and insignificant lack of fit (0.0736) indicated that reduced cubic model was highly significant and adequate to represent the actual relationship between the DE responses and the significant variables. A low pure error (0.77) indicating good reproducibility of the data was obtained. The standard deviation (STD) value of the model was 1.16, while the R^2^ and adjusted R^2^ of DE model were 0.9924 and 0.9823, respectively. The high values of R^2^ and adjusted R^2^, with low AAD values which showed a good agreement between the experimental results and predicted values by the reduced cubic model, expressed the goodness of model fit in this study.

### 2.4. Main Effects and Interactions between Parameters

The significant (*p* < 0.05) regression coefficients of the established model equation are listed in Table 4. Among them, X_2_X_3_ was not significant at the 95% level and their interaction was significant. This meant that the effect of X_2_ (enzyme loading) could be compensated by X_3_ (molar ratio) and vice versa. All the reaction parameters investigated positively affected DE, with X_2_ having the greatest effect, followed by X_1_ and X_3_. The interactions between these variables showed negative effects on DE. This was in agreement with a previously reported work which optimized the conditions of DE in a solvent system [24]. Most of the interactions between these variables were found to be significant at the 95% confidence level except X_1_X_2_.

**Figure 8:**
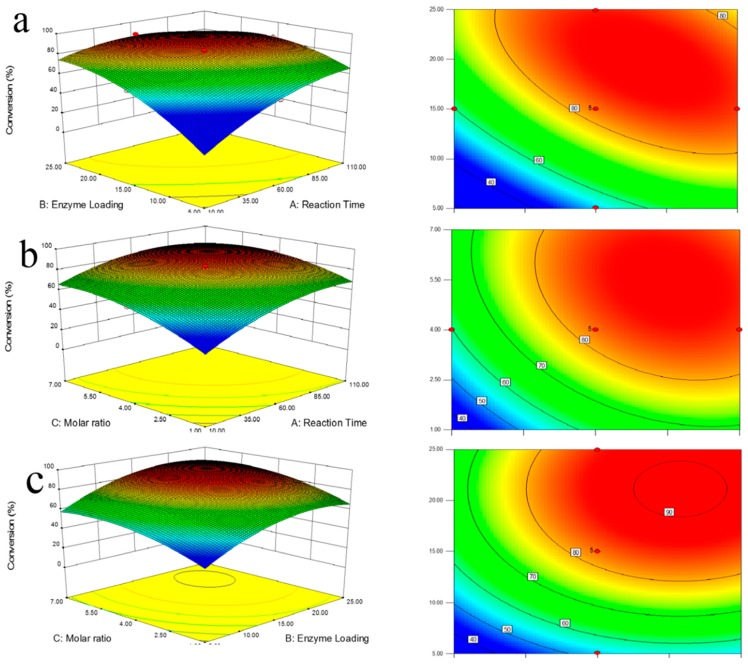
Response surface plot showing (**a**) the effect of reaction time, enzyme loading and their mutual effect on DE with molar ratio constant at 4; (**b**) the effect of reaction time, molar ratio and their mutual effect on DE with enzyme loading constant at 15%. (**c**) the effect of molar ratio, enzyme loading and their mutual effect on DE with reaction time constant at 60 min.

Figure 8a shows the response surface plots as a function of reaction time, enzyme loading and their mutual effects on the synthesis of ALA phytosterol esters. At the beginning of the reaction (10 min), DE showed a linear increase in enzymatic synthesis yield when the enzyme loading was increased. This suggested that in the range of enzyme loadings we designed, the more enzyme loaded the higher of initial esterification rate achieved. However, with longer time (110 min), the 3D surface plot showed maximal DE when moderate amounts of enzyme (15.4%) were used in the reaction and DE decreased when more enzyme was loaded. The decrease in DE when large amounts of lipases were employed can be explained by the fact that some lipase may become agglomerated and the contact area between substrate and lipase consequently reduced. [38,39,40]. Figure 8b depicts the response surface plots as a function of molar ratio versus enzyme loading at a reaction time of 60 min. Higher DE was observed with an increase in molar ratio and enzyme loading until it reached the equilibrium point at 5.5 molar ratio and 15.4% enzyme loading. Figure 8c show a the effect of molar ratio and reaction time on DE with the amount of enzyme held constant at 15.4%. DE increased with an increase of reaction time and molar ratio. The DE, however, decreased slightly at a molar ratio of approximately 6. When the molar ratio was greater than 6, it meant too much ALA was added, the enzyme and phytosterols were diluted causing a lower degree of esterification.

### 2.5. Optimization and Model Verification

Models of the optimal conditions for the lipase-catalyzed esterification synthesis of phytosterol esters with ALA were predicted using the optimization function of the Design Expert software. All the variables were set in the range of the design to obtain the maximum DE. The most desirable reaction conditions for the optimum DE were as follows: 69.2 min for reaction time, 20.33% for enzyme loading and 5.6 for molar ratio of ALA to phytosterols. The predicted DE under these conditions was 91.25%. To verify the predicted results of this model, an experiment was conducted under the optimum conditions and 90.31 ± 1.62% (*n* = 3) DE was obtained. The good correlation between the actual and predicted value verified the validity of the response model and the optimal point obtained. In the solvent-free system, excess ALA was used to reduce the system viscosity and improve the DE of phytosterols. After the reaction, the excess ALA was removed by washing the product twice with hot water, and absolute ethyl alcohol eight times. Obviously, the immobilization of CRL on HMSS-C_18_ was a highly efficient method for esterifying phytosterols with ALA.

**Figure 9:**
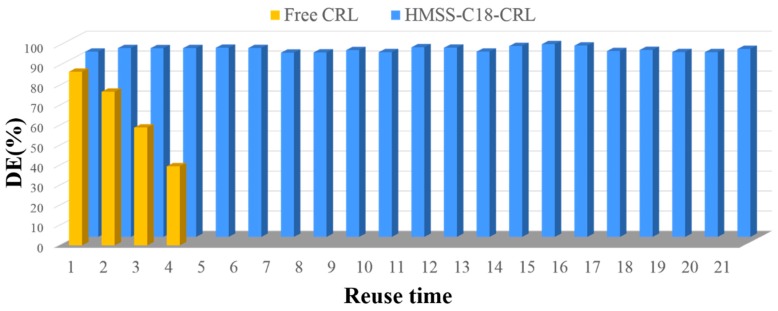
Reuse of free CRL and HMSS-C_18_-CRL for esterification of phytosterols in solvent-free system (reaction conditions: 5.5 molar ratio of ALA to phytosterol, 15.4% for enzyme loading, 50 °C, 250 rpm, 92 min).

The reusability of the immobilized enzyme is crucial for practical applications. The reuse of CRL powder and CRL immobilized on HMSS-C_18_ was evaluated using the esterification of phytosterols with ALA at the optimal conditions obtained from RSM in a batch reactor, namely 70 min for reaction time, 20% for enzyme loading and 5.6 for the molar ratio of ALA to phytosterol. At the end of each batch, the CRL powder and HMSS-C_18_-CRL were removed from the reaction medium and reused without any processing. In Figure 9, the degree of esterification using CRL powder dropped rapidly with repeated use. In contrast to this, the degree of esterification of the immobilized one could still reach 90.5% of its first value after 10 uses. The results confirmed that the CRL immobilized on HMSS-C_18_ has a good reaction condition durability and immobilization has a positive influence which is due to the micro-environment provided by the carrier. In the synthesis of phytosterols esters of ALA, the solvent-free system was applied successfully with immobilized CRL with a high degree of esterification in a short time and the system could be reused for at least ten times without any significant decrease in the degree of esterification.

## 3. Materials and Methods

### 3.1. Materials

*Candida rugosa* lipase (lyophilized powder, Type VII, 700 U/mg solid), trimethoxypropylsilane (98%), trimethoxyphenylsilane (98%) trimethoxyoctylsilane (96%), trimethoxyoctadecylsilane (90%), and *p*-nitrophenylpalmitate (p-NPP) were purchased from Sigma-Aldrich (St. Louis, MO, USA). Phytosterols (β-sitosterol 77%, campesterol 17%, stigmasterol 5%) was purchased from Xi’an Bluesky Biological Enineering Co. Ltd. (Xi’an, China). α-Linolenic acid (80%), was purchased from Henan Linuo Biochemical Co. Ltd. (Anyang, China). The BCA Protein Assay Kit was purchased from Beyotime Biotechnology (Shanghai, China). Toluene, triethylamine and hexadeyl-trimethylammonium bromide were purchased from Sinopharm Chemical Reagent (Shanghai, China) and were of analytical reagent grade.

### 3.2. Synthesis of HMSS and Different Modified HMSS

HMSS were prepared and purified according to previously reported methods [14,34,41]. The modification of HMSS was performed by adding 1 g of HMSS, 2.5 mM of silane coupling agent and 33 μL triethylamine into 40 mL toluene [42]. The mixture was placed in a Teflon-lined stainless-steel autoclave (130 °C) for 20 h. The product was collected by centrifugation (8000 rpm, 8 min), followed by washing with ethanol several times. The HMSS modified with propyl-(C_3_), phenyl-(C_6_), octyl-(C_8_), octadecyl-(C_18_) were denoted as HMSS-C_3_, HMSS-C_6_, HMSS-C_8_, and HMSS-C_18_, respectively.

### 3.3. Immobilization of CRL on HMSS with Different Surface Modifications

First, 1.5 g CRL was suspended in 30 mL of sodium phosphate buffer solution (50 mM, pH 7.0). Enzyme suspension was stirred at 4 °C for 30 min at 200 rpm and then centrifuged. The supernatant was taken, and the protein concentration was determined by a BCA protein assay. The supernatant (30 mL) was added to flasks containing 1 g of HMSS or individual modified HMSS, which were previously pre-wetted in ethanol and washed with 30 mL of buffer solution. The mixture was placed in a shaking incubator at 30 °C and 200 rpm, while continuously shaking for 30 min to finish immobilization of CRL. The immobilized CRL was separated from the enzyme solution by filtration and washed with phosphate buffer (50 mM, pH 7.0) three times to remove any unbound enzyme. The supernatant was collected to assay the amount of residual protein concentration. The resulting immobilized CRL was lyophilized and stored at 4 °C prior to use.

### 3.4. Lipase Activity Assay

The immobilized CRL activity was measured by the detection at 410 nm on a UV/VIS spectrophotometer of the *p*-nitrophenol (*p*-NP) resulting from the enzymatic hydrolysis of *p*-nitrophenyl palmitate (*p*-NPP) in PBS (50 mM, pH 7.0) for 5 min in shaker (160 rpm). In order to find the best reaction temperature for free lipase and immobilized lipase, experiments were conducted at different temperatures (20–70°C). One unit (U) of enzyme activity was defined as the amount of enzyme that hydrolyzes 1 μmol of *p*-NPP per minute under the conditions described previously. Finally, the most effective immobilized enzyme was chosen for the catalysis study and response surface methodology experiments. The thermal stabilty test of HMSS-C_18_-CRL and free CRL was performed with the *p*-NPP method by incubation for 30 min in the absence of substrate at different temperatures (20–70°C) and the residual activities was measured. Residual activities were calculated as the ratio of the activity of enzyme measured incubation to the maximal activity of the enzyme.

### 3.5. Characterization

N_2_ adsorption-desorption analysis was measured on a Beckman Coulter SA 3100 plus surface area analyzer (Beckman Coulter, CA, USA) at 77K. The surface area was evaluated from the adsorption branch in the relative pressure range of 0.05–0.15 using the Brunauer-Emmett-Teller (BET) method. The surface morphologies of the samples were observed by scanning electron microscopy (SEM; SU8010, Hitachi, Tokyo, Japan) at 200 kV and transmission electron microscopy (TEM; TECNAI G2 20S-TWIN, Hillsboro, OR, USA). Fourier transform infrared (FT-IR) spectra were recorded on a TENSOR 27 FTIR spectrometer (Bruker, Karlsruhe, Germany). The UV-vis spectra were recorded using a DU 800 UV-vis spectrometer (Beckman Coulter, CA, USA).

### 3.6. Effect of Temperature

The reaction temperature affects not only the esterification rate, but also the stability and the activity of enzyme. A preliminary study was conducted prior to the RSM work to examine the effect of temperature. The effect of the temperature on the synthesis of phytosterols ester in solvent free system was investigated as time course of the reaction. The range of temperatures tested was between 40 °C and 70 °C. For these trials, the enzyme loading and molar of phytosterols to ALA were kept at 15% (based on the weight of phytosterols) and 1:4, respectively. The effect of substrate molar ratio (1:2–1:6) and enzyme loading (5%–25%, to the weight of total substrate *w*/*w*) on DE were investigated under the identical method [43].

### 3.7. Response Surface Methodology

A five-level, three factorial central composite rotatable design (CCRD) was employed to study the response, namely, the esterification degree of phytosterols. The independent variables and their levels selected were as follows: reaction time (T: 10–110 min), enzyme load (E: 5–25%), and substrate molar ratio of ALA to phytosterols (M: 1:1–7:1). A CCRD consists of three parts: Factorial points (−1, 1), center points (0, 0), and axial points (−1.68, 1.68). Each variable to be optimized was coded at these levels, and the ranges were shown in Table 4. The data from the experiments performed were analyzed using Design Expert 8.0.5b, then interpreted in three main analytical steps: ANOVA, a regression analysis and the plotting of response surface were performed to establish an optimum condition for the esterification reaction. The level of significance for all tests was set at 95% confidence level. The goodness of the models established was determined using the coefficient of determination (R^2^) together with standard deviation (STD) values and ANOVA. The 3D surface plots were developed using the fitted reduced cubic (modified model) polynomial equations with backward elimination obtained by holding two of the independent variables at a constant value and changing the levels of the other two variables.

### 3.8. Qualitative and Quantitative Analysis of Phytosterols Esters

The qualitative and quantitative analysis of phytosterols esters were conducted with a previous reported method [30]. An Agilent 6890 Series II gas chromatograph (Hewlett–Packard Co., Avondale, PA, USA), equipped with a FID and a fused silica capillary column (DB-5 HT, 15.0 m × 0.32 mm × 0.10 mm, Agilent Technologies, Deerfield, IL, USA) was used. The carrier gas was nitrogen and the total gas flow rate was 3.5 mL/min. The injector and detector temperatures were maintained at 320 °C and 350 °C, respectively. The oven temperature was held at 210 °C for 2.0 min, then increased to 320 °C at a rate of 10 °C /min and held for 15 min, then increased to 380 °C at a rate of 10 °C /min, finally it was held at 380 °C for another 5 min. The injection volume was 1 μL in split mode. The split ratio was 50:1. The degree of esterification (%) of phytosterols was calculated from the GC profile of reactants using Equation (1) [42]:

(2) $$\mathrm{Degree}\;\mathrm{of}\;\mathrm{Esterification}\;\left(\mathrm{DE}\right)=\frac{B}{B+1.63\times A}\times 100\%$$

where A = peak area of total phytosterols; B = peak area of total phytosterols esters. 1.63 = ratio of molecular weight of total phytosterols esters of ALA to molecular weight of total phytosterols.

## 4. Conclusions

The immobilization of CRL onto modified HMSS with different hydrophobicity has been studied and the immobilized lipase showed improved enzymatic activities with increasing hydrocarbon chain length. A solvent-free system for the synthesis of phytosterol esters was prepared using the immobilized CRL. RSM was successfully applied to model and optimize the conditions used in the esterification reaction and the optimized process variables were reproducible. Benefitting from an appropriate pore size and large cavity volume, and most importantly the hydrophobic microenvironment, the HMSS-C_18_-CRL showed exceptional performance in the catalytic reaction and excellent recycle ability for 21 times with DE of more than 90% under the optimized conditions: 70 min for reaction time, 20% enzyme loading and 5.6 molar ratio of ALA to phytosterol. This investigation opens new opportunities for developing HMSS-C_18_ as an ideal support for enzyme immobilization and as a biocatalyst for natural food production.

## Figures and Tables

**Table 1:** Textual parameters of HMSS-C_18_ and HMSS0C_18_-CRL.

Sample	Surface Area (m^2^/g)	Pore Volume (cm^3^/g)	Pore Size (nm)
HMSS-C_18_	399	1.49	12.0
HMSS-C_18_-CRL	260	1.06	11.9

**Table 2:** Central composite design arrangement, experimental value and predicted value obtained from reduced cubic model with backward elimination for the CRL@C_18_@HMSS catalyzed esterification to synthesize phytosterols esters of ALA.

Run	Independent Variables			Responses	
	E ^a^	T ^a^	M ^a^	Experimental	Predicted
1	−1	−1	−1	42.96 ± 3.04 ^b^	42.49
2	1	−1	−1	67.40 ± 2.71	66.83
3	−1	1	−1	68.36 ± 0.41	67.88
4	1	1	−1	80.33 ± 0.15	79.75
5	−1	−1	1	57.75 ± 2.58	57.94
6	1	−1	1	79.69 ± 0.06	79.78
7	−1	1	1	85.26 ± 0.44	85.45
8	1	1	1	86.72 ± 0.16	86.80
9	−1.68	0	0	59.66 ± 0.59	59.82
10	1.68	0	0	84.40 ± 0.27	84.79
11	0	−1.68	0	50.85 ± 8.72	51.13
12	0	1.68	0	87.28 ± 0.04	87.55
13	0	0	−1.68	60.78 ± 9.21	61.84
14	0	0	1.68	84.64 ± 0.42	84.13
15	0	0	0	83.05±0.13	82.74
16	0	0	0	81.56 ± 0.41	82.74
17	0	0	0	82.86 ± 0.24	82.74
18	0	0	0	82.39 ± 0.27	82.74
19	0	0	0	83.95 ± 0.08	82.74

^a^ E = enzyme loading; T = reaction time; M = substrate molar ratio (ALA to phytosterols). ^b^ Mean ± SD, *n* = 2.

**Table 3:** ANOVA table of the response surface reduced cubic model.

	Sum of Squares	Degrees of Freedom	Mean Square	F Value	*p*-Value
Model	3357.74	11	305.25	355.18	<0.0001
Residual	6.02	7	0.86		
Lack of Fit	2.92	3	0.97	1.26	0.3999
Pure Error	3.09	4	0.77		

R^2^ = 0.9982; AAD = 0.1189.

**Table 4:** Independent variables and their levels for central composite design in optimization of lipase-catalyzed esterification process.

Independent Variables	Symbol	Coded Variable Levels				
		−1.68	−1	0	1	1.68
Reaction time (min)	T	10.0	30.3	60.0	89.7	110.0
Enzyme load (%)	E	5.0	9.1	15.0	20.9	25.0
Molar ratio	M	1.0	2.2	4.0	5.8	7.0
